# The decline of iodine therapy in the treatment of Graves’ disease in a hospital center: a 20-year analysis

**DOI:** 10.20945/2359-4292-2026-0013

**Published:** 2026-02-16

**Authors:** Isabela Busto Silva, Gisah Amaral de Carvalho, Fabíola Yukiko Miasaki, João Pedro Ignotti de Almeida, Matheus Pessini Sousa, Caio Pereira Mueller, Hans Graf, Mariana Driesel Bertolin, Cléo Otaviano Mesa Júnior

**Affiliations:** 1 Departamento de Medicina Interna, Serviço de Endocrinologia e Metabologia (SEMPR), Universidade Federal do Paraná, Curitiba, PR, Brasil; 2 Universidade Federal do Paraná, Curitiba, PR, Brasil

**Keywords:** Hyperthyroidism, Graves’ disease, radioiodine

## Abstract

**Objective:**

To evaluate and describe the changes in the therapeutic approach to Graves’ disease at a tertiary hospital center over a 20-year period, with an emphasis on the frequency of prescription and the timing of radioactive iodine indication.

**Subjects and methods:**

We conducted a retrospective analysis of data from medical records of patients recently diagnosed with Graves’ disease (GD) and followed up at a single institution during two consecutive periods: Group A diagnosed between 2002 and 2010, and Group B between 2011 and 2022. We analyzed the percentage of patients who underwent iodine therapy and were considered to have failed therapy if they did not achieve hypothyroidism or euthyroidism, comparing the results between both groups.

**Results:**

A total of 597 GD patients were included, of which 223 underwent radioactive iodine (RAI) therapy (37.35%). In Group A, 176 patients (64%) received RAI treatment, whereas, in Group B, only 47 patients were given this therapeutic indication (14.6%) (p < 0.001). The reduction in RAI prescriptions between both periods was independent of the therapeutic indication. Interestingly, RAI prescription due to relapse after clinical treatment was uncommon in both study groups. There was a significant increase in the duration of antithyroid drug (ATD) therapy before RAI prescription in Group B compared to Group A.

**Conclusion:**

Significant changes were observed in GD treatment, with a decline in the use of RAI as a first-line or salvage therapy. Nonetheless, radioiodine therapy remained an effective and safe treatment modality with successful cure rates.

## INTRODUCTION

Graves’ disease (GD), the most common cause of hyperthyroidism, is associated with significant comorbidities and reduced quality of life ^(1)^. For over 70 years, three primary treatment options have been available: antithyroid drugs (ATD), radioactive iodine (RAI), and thyroidectomy. Preferences for first-line treatment vary across countries and regions, evolving over time based on new studies, updated evidence-based guidelines, or shifts in expert opinion ^(2)^. Thus, selecting the most suitable treatment for patients necessitates thoughtful discussions and active collaboration in decision-making ^(3)^.

Radioactive iodine is an effective treatment for managing hyperthyroidism in patients with GD, as iodine-131 is rapidly absorbed by the thyroid, with its beta emissions causing localized tissue destruction. Therapy is considered simple, safe, and often the most cost-effective option, offering rapid and definitive control of excess thyroid hormone effects ^(4)^. The ultimate goal of RAI therapy, as delineated in current American Thyroid Association guidelines, is to achieve near-complete ablation of the thyroid gland, leading to permanent hypothyroidism as the intended outcome ^(5)^.

In recent decades, there has been a noticeable shift in the strategies and trends regarding the use of RAI compared to ATD ^(6)^, particularly in the United States, where RAI therapy has been traditionally favored. A 2011 survey showed that over half of US clinicians preferred RAI, whereas around 40% opted for initiating a one- to two-year course of ATD ^(7)^. However, by 2023, preferences had significantly shifted, with 91.5% of respondents favoring ATD and only 7% continuing to prefer RAI ^(8)^.

To further understand the evolving landscape of GD management, we conducted a study at our center to analyze current practices involving RAI and evaluate trends over the past 20 years. Our research aimed to assess key variables, including RAI prescription rates, prescribed activity levels, intervals between ATD use and RAI administration, and the effectiveness of this treatment modality. We then compared these variables across two decades to identify significant trends and changes in practice.

## SUBJECTS AND METHODS

Our study is a retrospective analysis of medical records from patients diagnosed with GD and followed at a single institution during two distinct periods: Group A, consisting of patients diagnosed between 2002 and 2010, and Group B, comprising those diagnosed between 2011 and 2022. Diagnosis was based on suppressed thyroid-stimulating hormone (TSH) levels, elevated free thyroxine (FT4) or total T3 levels (TT3), positive thyrotropin receptor antibodies (TRAb), and/or thyroid scintigraphy showing diffuse increased tracer accumulation in an enlarged thyroid gland. As laboratory analyses were modified due to the extended follow-up period, adjustments to the methodological approach were necessary. Hence, TSH, FT4, and TT3 levels were measured with chemiluminescence assays (ABBOTT).

From July 2019 onward, analyses were conducted using the ALINITY system (TSH 0.35–4.94 mIU/mL; FT4 0.7–1.48 ng/dL; TT3 64–152 ng/dL). Between 2011 and July 2019, the ARCHITECT system was used (TSH: 0.4–4.5 IU/mL; FT4: 0.7–1.8 ng/dL; TT3: 80–180 ng/dL). Before 2011, TSH and thyroid hormones were measured using a microparticle enzyme immunoassay with the AXSYM system, also by ABBOTT (TSH: 0.4–4.0 IU/mL; FT4: 0.7–1.9 ng/dL; TT3: 80–200 ng/dL). The TRAb assay underwent several changes: from 2002 to 2005, it was performed using a first-generation radioimmunoassay, known as the TRAk human radioimmunoassay (BRAHMS Diagnostica, Germany), with results expressed as a percentage of inhibition of TSH binding, considering values ≥10% positive. Afterward, the TRAb assay was performed using chemiluminescence, deemed positive when levels exceeded 0.55 IU/L. From July 2019 onward, the TRAb assay method changed to using the Immulite 2000 equipment (Siemens, USA), with levels exceeding 1.5 IU/L considered positive.

We evaluated the percentage of patients undergoing iodine therapy who were deemed to have failed therapy if they did not achieve hypothyroidism or euthyroidism. Analyzed variables for this group included age, sex, smoking status, presence of thyroid eye disease (TED), therapeutic efficacy (defined as achieving hypothyroidism or euthyroidism within one year without the need for additional therapy), prescribed activity, maximum ATD dose, duration of ATD use before radioiodine administration, and time to progression to hypothyroidism. The RAI dose was empirically adjusted for each patient based on disease severity and goiter size, typically between 15–25 mCi, without a standardized protocol but rather through individualized clinical evaluation. Standard practice includes administering oral glucocorticoid prophylaxis (prednisone 0.5 mg/kg/day for 2–3 weeks, tapered thereafter) to all patients with active thyroid orbitopathy, those with moderate to severe inactive thyroid orbitopathy, and smokers with TRAb levels >3 times the upper limit of normal, even in the absence of evident orbital disease.

Data were analyzed using the IBM SPSS Statistics (v. 29.0) software. Associations between categorical variables were assessed using Fisher’s exact test or the Chi-square test. Comparisons between both periods defined by the year of diagnosis (before 2010 or after) for quantitative variables employed the non-parametric Mann-Whitney test. The normality of continuous variables was evaluated using the Kolmogorov-Smirnov test, and *p*-values below 0.05 were deemed statistically significant.

## RESULTS

**Figure 1:**
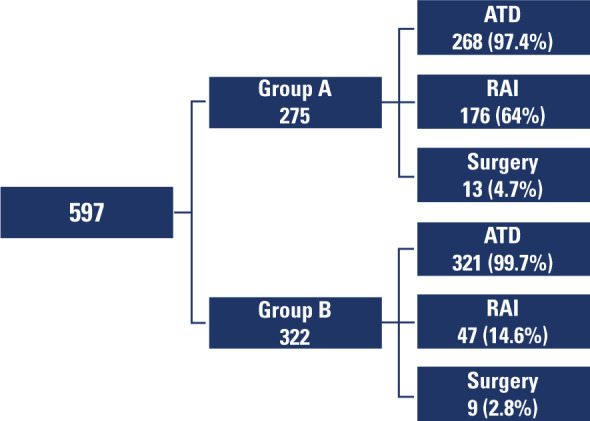
Flowchart illustrating the management of patients with Graves’ disease and the therapeutic modalities indicated.

A total of 597 patients with GD were included in the analysis, with 223 (37.35%) of them undergoing RAI treatment. Among these, 275 were diagnosed with GD between 2002 and 2010 (Group A), and 322 were diagnosed between 2010 and 2022 (Group B) (**Figure 1**). In Group A, 176 patients (64%) received RAI treatment, whereas in Group B, only 47 patients (14.6%) underwent this therapy (*p* < 0.001).

The baseline characteristics of both groups were generally similar, except for a higher prevalence of TED in Group A (**Table 1**). Due to changes in methodology and low testing rates at the beginning of the study, it was not possible to compare TRAb values between groups. The median TRAb values were 11.6 and 12.2 in Groups A and B, respectively. The sample was divided into two consecutive periods due to the notable decline in RAI therapy prescriptions over time. Furthermore, the increasing number of studies published in the last decade suggesting the safety of prolonged ATD use may have impacted clinical practice, supporting the rationale for analyzing the periods separately.

**Table 1.** Baseline characteristics of participants undergoing radioactive iodine treatment

Variable	Group A (2002–2010)	Group B (2011–2022)	p-value
Patients (n)	176	47	–
Age (mean)	39.4	39.1	0.898
Female sex (%)	147 (83.5%)	43 (91.5%)	0.247
Active smoking (%)	63 (41.7%)	8 (21.1%)	0.062
Graves’ ophthalmopathy (%)	90 (51.1%)	12 (25.5%)	0.002
TRAb			
Not tested	152 (48.4%)	15 (31.9%)	–
Positive	16 (9.1%)	28 (59.6%)	–
Negative	8 (4.5%)	4 (8.5%)	–
General follow up in years (median/standard deviation)*	8/5.1	6 / 2.8	< 0.001

TRAb: Positive thyrotropin receptor antibodies.

* 5 missing data on status or year of follow-up completion.

**Figure 2:**
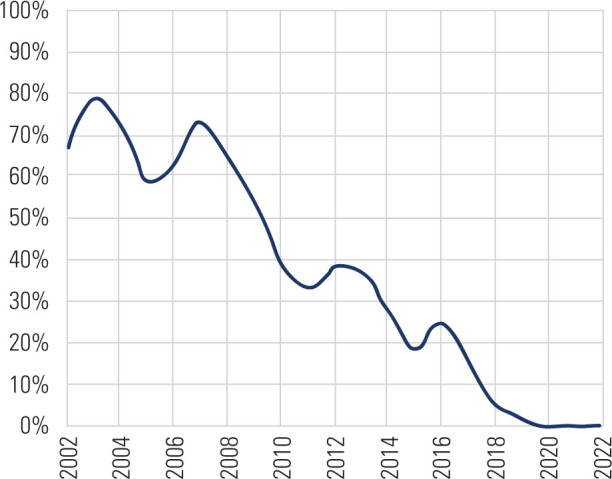
Percentage of patients with Graves’ disease undergoing radioactive iodine treatment from 2002 to 2022.

Our findings also revealed that RAI treatment prescription for GD has significantly declined in recent years, and this occurred consistently between both periods analyzed, regardless of the therapeutic indication (**Figure 2**).

**Table 2** outlines the reasons for prescribing RAI in both groups, with the primary reason being treatment failure with ATD, which accounted for 57% during the earlier period (2002–2010) and 13.3% during the later period (2010–2022). Interestingly, relapses after clinical treatment were an uncommon reason for RAI prescription in both groups. Similarly, RAI as a first-line therapeutic option was infrequently observed across both periods and RAI following thyroidectomy was only observed in Group A (1.09%).

**Table 2.** Indication for radioactive iodine treatment in patients with Graves’ disease

RAI indication	Total n (%)	2002–2010 n (%)	2010–2022 n (%)
Received RAI indication	223 (37.36%)	176 (64%)	47 (14.6%)
Initial option	11 (1.84%)	9 (3.27%)	2 (0.62%)
Relapses after ATD treatment	9 (1.5%)	7 (2.5%)	2 (0.62%)
ATD treatment failure	200 (33.5%)	157 (57%)	43 (13.3%)
After thyroidectomy	3 (0.5%)	3 (1.09%)	0 (0%)
Total number of patients with GD	597 (100%)	275 (100%)	322 (100%)

RAI: radioactive iodine treatment; ATD: antithyroid drug; GD: Graves’ disease.

Among the analyzed variables, the maximum methimazole dose did not differ significantly between groups, with a mean dose of 30.4 mg (SD ±13.3) in Group A and 30.8 mg (SD ±13.1) in Group B (*p* = 0.643). Nevertheless, the duration of methimazole treatment was significantly longer in Group B, with a median duration of 37.7 months (±33.7) compared to 23.2 months (±24.7) in Group A (*p* = 0.002).

The prescribed RAI activity was similar between groups, with a mean dose of 23.1 mCi (SD ±5.2) in Group A and 23.6 mCi (SD ±6.0) in Group B (*p* = 0.428). The necessity for additional RAI doses occurred in 12.3% of patients in Group A and 13.6% in Group B, indicating no significant difference (*p* = 0.801). Notably, treatment effectiveness was significantly higher in Group A, as 89.5% of patients achieved hypothyroidism or euthyroidism compared to 69.6% in Group B (*p* = 0.002). The median time to hypothyroidism following RAI therapy was identical for both groups at 4 months (*p* = 0.642). The prevalence of patients with TED undergoing RAI differed significantly between both groups. In Group A, 90 patients (51.1%) had TED, whereas only 12 patients (25.5%) had the diagnosis (*p* = 0.002) in Group B.

## DISCUSSION

This study analyzed a cohort of nearly 600 patients with GD treated at a single institution over a 20-year period, with a particular focus on RAI therapy. The primary finding was a notable decrease in the utilization of RAI for treating GD, a trend that significantly accelerated over the last decade (**Figure 2**). Despite RAI’s long-standing reputation as a straightforward, cost-effective, and rapid therapeutic option, its prescription has progressively declined.

Although we did not seek to identify the factors behind this shift, several plausible explanations exist. Concerns over the risk of developing new or worsening TED have likely played a role, as has evidence suggesting that prolonged ATD therapy may increase the likelihood of achieving remission in GD. Consequently, indications for RAI have gradually diminished in recent years, a stark contrast to the past when it was routinely recommended for patients who remained hyperthyroid after two years of ATD therapy. This trend also mirrors efforts to prevent permanent hypothyroidism and a move towards long-term medical management. In line with this shift, patients in our study who later transitioned to RAI had undergone ATD therapy for longer periods. Therefore, contemporary RAI candidates typically present with more severe diseases. Our analysis revealed these patients had significantly higher maximum methimazole doses compared to those receiving ATD therapy alone (30.4 vs. 24.3 mg; *p* < 0.001). Our findings align with research indicating ATD patients tend to be younger, possess fewer comorbidities, and include pregnant individuals, thereby reinforcing the preference for ATDs in these groups and ^(9)^, thereby reinforcing the reserved use of RAI for more challenging cases.

In our study, iodine therapy was initially chosen for a small proportion of patients in both groups (i.e., 3.3% in Group A and 0.6% in Group B). These rates are significantly lower than the 14.1% reported in a 2013 European survey and well below the 59% observed in a 2011 United States survey ^(7,10)^. Intriguingly, a recent international survey of clinical practice patterns published in 2024 ^(8)^, which garnered responses from endocrinologists across 85 countries, revealed a striking shift in the preference for RAI as primary therapy. Only 7.0% of respondents (95/1353) indicated RAI as their first-line treatment for GD, revealing a significant regional variation ranging from a minimum of 1.8% to a maximum of 13.1% and highlighting a global trend away from RAI as an initial treatment option. The treatment trends identified in this study deviate from the 2016 American Thyroid Association hyperthyroidism practice guideline, which recommends an average RAI dose of 10–15 mCi ^(5)^. These findings also contrast with a meta-analysis and systematic review that reported a mean RAI dose of 8.5 mCi (range 6.8–12.6) ^(11)^. In our analysis, RAI doses were consistently higher, exceeding 20 mCi in both groups.

This study provides a basis for comparison with a previous study conducted at the same institution, by Sztal-Mazer and cols. ^(12)^, which analyzed cases diagnosed between 1994 and 2009. During that period, the average initial RAI dose was 21.4 mCi, achieving an overall success rate of 86%. The authors concluded that higher doses were associated with more successful treatment outcomes and earlier achievement of therapeutic goals. Conversely, despite similar RAI doses across groups, we found significant differences in success rates (89.5% in Group A vs. 69.6% in Group B). This suggests that RAI is increasingly being reserved for patients with more severe illnesses. A meta-analysis of randomized controlled trials revealed an average cure rate of hyperthyroidism with RAI treatment of 77.8% ^(13)^. Nevertheless, predicting the timing for RAI effects poses challenges, complicating patient monitoring. Our findings are consistent with the association between higher RAI doses and shorter remission times, evidenced by our study’s median time to hypothyroidism being 4 months—shorter than the 6-month median observed in other studies ^(14)^. The median time to hypothyroidism in our study was calculated from the first post-RAI therapy assessments, potentially introducing a bias due to the assessment timing and confirmation of outcomes. Numerous factors, including gland size, volume, administered dose, and the presence of positive anti-thyroid peroxidase antibodies, likely influence these outcomes ^(15)^. Further research should explore these variables to enhance the efficacy and monitoring strategies of RAI treatment.

Despite our promising findings, some limitations must be acknowledged, such as its retrospective design which restricts the ability to pinpoint factors responsible for the observed reduction in RAI prescriptions precisely. Additionally, samples were divided based on the year of diagnosis to investigate changes in diagnostic practices; as a result, Group A had an earlier diagnosis and consequently a longer follow-up time, which significantly limits our study as it may have impacted observed outcomes. Other limitations include potential data collection biases and challenges in determining causal relationships. Regardless, these drawbacks were crucial for interpreting results and crucial for validating the conclusions. Additionally, the TRAb test was conducted less frequently in Group A compared to Group B due to limited availability of testing methods during the earlier period. Nevertheless, these limitations, our study’s primary finding remains unchanged: a significant decrease in the prescription of RAI for treating GD over the past two decades. The scarcity of large-scale Brazilian studies on changes in GD treatment practices over time further underscores the relevance of our findings, which reflect trends at a single reference center over a 20-year period and may not represent nationwide practices in Brazil.

In conclusion, our results demonstrate a significant decline in the use of iodine therapy for GD in recent years. Despite this decline, RAI therapy continues to be an effective and safe option, demonstrating high success rates in treating hyperthyroidism. The reasons behind this reduction are not fully understood, and contributing factors may include concerns about inducing hypothyroidism, the potential for worsening TED, and increased confidence in the long-term use of ATD. Future studies should explore the benefits and drawbacks of different therapeutic modalities for GD, with an emphasis on aspects such as mortality reduction, quality of life improvements, and other long-term outcomes. It is crucial to assess whether the growing preference for ATD as a primary treatment indeed offers superior benefits for GD patients.

## Data Availability

datasets related to this article will be available upon request to the corresponding author.
